# Metropolitan Hospital Sunday Fund

**Published:** 1910-12-03

**Authors:** 


### METROPOLITAN HOSPITAL SUNDAY FUND.

A meeting of the Council of this Fund was held at the Mansion House on Wednesday, November 30, under the Presidency of the Right Hon. the Lord Mayor (Sir Vezey Strong).

Sir John Bell moved "that the Report of the Council be, and is hereby, received and adopted, and is ordered to be published." In doing so he remarked that he was sure all present would have read with delight that, owing to the efforts which had been made, a larger collection had been realised this year than last. Clergymen and ministers of all denominations had put their shoulders to the wheel and done their best to assist the great cause. Sir Dyce Duckworth seconded the motion, which was carried.

The Lord Mayor, Sir Eardley Wilmot, and the Rev. Mr. Arnot, were elected to fill vacancies on the Council.

### GRANTS FOR SURGICAL APPLIANCES.

The Archdeacon of London proposed that the recommendation of the General Purposes Committee that the percentage of the Fund allocated to the purchase of surgical appliances be raised from 5 per cent. to 7½ per cent. be and is hereby adopted, and that the constituents be recommended to alter the Law and Constitution accordingly." In explanation of the resolution, he said that the proportion of 5 per cent. was not sufficient in the present day of surgical advance and operative skill ; and that was borne out by his experience in connection with the Surgical Aid Society. The Secretary of the Fund had for some time past exceeded the 5 per cent. allowed by the laws, and the present resolution would obviate the necessity of Sir Edmund Currie asking the Council each time to ratify his action in the matter.

The resolution was seconded by Mr. Hampton Hale and agreed to.

On the proposition of Mr. Hampton Hale, seconded by Sir Joseph Dimsdale, the Secretary of the Fund, Sir Edmund Hay Currie, was granted an honorarium of £100 to enable him to take a holiday for the restoration of his health after his recent attack of bronchitis.

The Secretary expressed his warm acknowledgments.

### GRANT TO A TESTIMONIAL.

On the application of Mr. Hampton Hale, the Council agreed to contribute from the Fund the sum of £20 towards a testimonial to Mr. Thomas Cole, of Bethnal Green, a man in poor circumstances who for a number of years had been connected closely with the Christian Evidence Society, Victoria Park, in which the Bishop of London was interested, and who had been instrumental in collecting a large sum for the Fund. The testimonial was necessary to relieve pressing needs.

### SUNDAY PICTURE SHOWS.

The Archdeacon of London reported that the Committee appointed to consider this subject had held several meetings, and were on the way to arriving at a decision, but that they could not report until an appeal which was now pending from the Bermondsey Council against the London County Council was decided. The question raised was as to whether the law was not circumvented by taking money in another way than for admission.

The meeting closed with thanks to the Lord Mayor for presiding, and congratulations on his attaining to his distinguished position.

